# Motor Effects of Intervention With Transcranial Direct Current Stimulation for Physiotherapy Treatment in Children With Cerebral Palsy: Protocol for a Randomized Clinical Trial

**DOI:** 10.2196/52922

**Published:** 2024-04-30

**Authors:** Anna Izabel Cangussu, Beatriz Lucarini, Igor de Freitas Melo, Paula Araújo Diniz, Marisa Mancini, Bernardo de Mattos Viana, Marco Aurélio Romano-Silva, Débora Marques de Miranda

**Affiliations:** 1 Faculdade de Medicina da Universidade Federal de Minas Gerais Federal University of Minas Gerais Belo Horizonte Brazil; 2 Instituto Nacional de Ciência e Tecnologia de Medicina Molecular Universidade Federal de Minas Gerais Belo Horinzote Brazil; 3 Departament of Occupational Therapy Universidade Federal de Minas Gerais Belo Horizonte Brazil; 4 Instituto Nacional de Ciência e Tecnologia Neurotech R Universidade Federal de Minas Gerais Belo Horizonte Brazil; 5 Departamento de Pediatria Faculdade de Medicina Universidade Federal de Minas Gerais Belo Horizonte Brazil

**Keywords:** cerebral palsy, tDCS, motor, development, randomized clinical trial, RCT, clinical trial, randomized, transcranial direct current stimulation, stimulation, children, child, brain stimulation, physical therapy, quality of life, researchers, researcher, neurological injuries, injury, injuries, gait, patient, patients

## Abstract

**Background:**

Children diagnosed with cerebral palsy (CP) often experience various limitations, particularly in gross motor function and activities of daily living. Transcranial direct current stimulation (tDCS) is a noninvasive brain stimulation technique that has been used to improve movement, gross motor function, and activities of daily living.

**Objective:**

This study aims to evaluate the potential additional effects of physiotherapy combined with tDCS in children with CP in comparison with physiotherapy only.

**Methods:**

This is a 2-arm randomized controlled trial that will compare the effects of tDCS as an adjunctive treatment during rehabilitation sessions to rehabilitation without tDCS. Children with CP classified by the Gross Motor Function Classification System as levels I and II will be randomly assigned to either the sham + rehabilitation group or the tDCS + rehabilitation group. The primary outcome will be the motor skills assessed using the Gross Motor Function Measure domain E scores, and the secondary outcome will be the measurement scores of the children’s quality of life. The intervention will consist of a 10-day stimulation protocol with tDCS spread over 2 weeks, with stimulation or sham tDCS administered for 20 minutes at a frequency of 1 Hz, in combination with physiotherapy. Physical therapy exercises will be conducted in a circuit based on each child’s baseline Gross Motor Function Measure results. The participants’ changes will be evaluated and compared in both groups. Intervenient features will be tested.

**Results:**

Data collection is ongoing and is expected to be completed by January 2025. A homogeneous sample and clear outcomes may be a highlight of this protocol, which may allow us to understand the potential use of tDCS and for whom it should or should not be used.

**Conclusions:**

A study with good evidence and clear outcomes in children with CP might open an avenue for the potential best use of neurostimulation.

**Trial Registration:**

Brazilian Registry of Clinical Trials RBR-104h4s4y; https://tinyurl.com/47r3x2e4

**International Registered Report Identifier (IRRID):**

PRR1-10.2196/52922

## Introduction

Cerebral palsy (CP) is a prevalent cause of physical disability among children, with reported incidence rates ranging from 2.0 to 2.5 per 1000 live births in developed countries, and even higher in low and middle-income countries, reaching up to 7 per 1000 live births [1]. The treatment goals for CP primarily focus on improving motor function and promoting independence in activities of daily living, as well as enhancing quality of life (QoL) [2]. Despite the efforts made in this area, the heterogeneous clinical presentations and responses to interventions pose a challenge in generalizing information about the effectiveness of specific treatment methods [2]. Guidance from the Cochrane Collaboration in 2019 suggests there is a need to standardize the outcomes studied in interventions, to provide functional and informative results and guide the selection of the most effective interventions and an effective individualized treatment [3].

Children with CP have difficulties in balance, motor function, and movement coordination. Physical therapy is used to improve these disabilities. Transcranial direct current stimulation (tDCS) is a complementary intervention that has been shown to enhance balance and improve movement coordination and gross motor function in children with CP [2,4,5]. There is a difference in oscillation speed when associating tDCS with virtual reality [5]. De Almeida Carvalho Duarte et al [2] have shown that children with CP subjected to 5 sessions of treadmill training stimulated with tDCS for 2 weeks with stimulation in M1 for 20 minutes exhibited improvements in temporal functional mobility, such as gait. The results were still maintained 1 month after the intervention. Balance and functional performance seem improved in children with CP after 5 sessions of tDCS anodal stimulation of the motor cortex plus treadmill training for 2 consecutive weeks.

Just 1 session of tDCS realized in the M1 region for 20 minutes in a group of children with CP showed a momentary increase in cortex activation with a direct influence on motor control and an improvement in gait and balanced outcomes [6]. Permanent effects in the population submitted to tDCS with clinical effects were observed in promoting functional improvement in children with CP [2,4].

According to the previous results, we hypothesized that tDCS use might improve specific motor skills and positively impact QoL in a more homogeneous population with CP. This study was designed to examine the impact of the combination of tDCS and conventional rehabilitation exercises on the improvement of gross motor function in children with CP classified as level I and II in the Gross Motor Function Classification System (GMFCS). The primary outcome measure will be assessed using the scores of the Gross Motor Function Measure (GMFM) dimension E and the secondary outcome is the children’s QoL.

According to Castelli and Fazzi [7], rehabilitation is a complex process that aims to promote the best possible participation and QoL for the child and the family. Rehabilitation for children with CP must be timely, intensive, and family-centered [7]. Despite the importance of understanding the effects on children’s QoL, QoL is not commonly measured.

Castelli and Fazzi [7] recommend intensive rehabilitation with trained professionals and a multidisciplinary team focused on the child’s functional improvement. Rehabilitation is one of the main parts as it involves performing functional assessments, establishing a functional prognosis, constructing a rehabilitation plan, and promoting a multidisciplinary approach [7].

## Methods

This study is a single-blind randomized controlled trial (RCT). The participants will be blinded to the treatment allocation during the trial, but the researchers conducting this study will be aware of the treatment allocation. This study will conform to the CONSORT (Consolidated Standards of Reporting Trial) guidelines for nonpharmacological interventions. This study has been approved by the ethics committee in research.

### Recruitment

All patients with CP who meet the previously described criteria for inclusion will be invited to participate in this study which will be realized in the Molecular Medicine Center of the Universidad Federal de Minas Gerais in Brazil.

### Sample Size

The sample size was calculated using G* Power software (version 3.1; Universität Düsseldorf) [8]. Considering an effect size of 0.5, power of 0.95, and probability of type I error (α) of 5%, the total sample size should be 176 volunteers, with 2 groups of 88 volunteers. The impact of the intervention will be evaluated in the pilot phase to determine a more accurate estimate of the population sample size. The participants will be included from July 2023 to July 2024. After the end of the intervention, the data collected will be separated into groups, these being the intervention group (IG) and the control group (CG). We will carry out the analysis of the pre- and postintervention GMFM variables and the Pediatric Quality of Life Inventory (PedsQL). We will carry out the analysis between groups (CG and IG) and between individuals in the same group, and will compare participants with themselves, to quantify and describe the results. Analysis of covariance will be chosen to observe preexisting differences between groups, allowing for comparison of postintervention scores while controlling for baseline scores.

### Participants and Eligibility Criteria

The participants in this study will be recruited through advertisements at clinics and the intervention will take place at the Molecular Medicine Center of the Universidade Federal de Minas Gerais in Brazil. To be eligible, participants must have a medical diagnosis of CP, be classified as GMFCS I or II, be aged between 8 and 12 years, be able to walk independently, and be able to understand commands. Exclusion criteria include other neurological conditions, recent history of surgery, uncontrolled epilepsy, a cranial metallic implant, and the use of a hearing aid. The waiting list group will have access granted afterwards based on the evidence.

### Ethical Considerations

This study was evaluated and approved by the local ethical board at Universidade Federal de Minas Gerais (50504021.0.0000.5149). All participants and their guardians will be informed about the research protocols and will be required to sign the free and informed consent form and the free and clarified assent form. Participation will only be allowed when both the child and parent have agreed to participate. All data will be handled as anonymous. Following the national law, participation in the intervention must be voluntary and reimbursement will be available only for research-related transportation.

### Measures

#### Overview

Both instruments will be used with all participants before and after the interventions to assess whether there was a significant improvement in gross motor movement according to the E domain of the GMFM or in QoL.

#### About GMFCS

This is a scale used to classify the level of impairments of the gross motor function of the CP population [9]. GMFCS allows us to classify functionality about walking, sitting, and standing [8]. It classifies gross motor function into 5 levels, as follows: level I: no access; level II: walks with limitation; level III: walks using a manual mobility device (such as a Canadian crutch, walker, or cane); level IV: self-mobility with a limitation (such as using a regular or automatic wheelchair); level V: patient transported via manual wheelchair by a responsible person. Initially, the entire sample will be evaluated using this instrument [10].

#### About GMFM

This instrument evaluates quantitatively the change in gross motor function in children with CP. The evaluation has five dimensions: dimension A, bed and roll; dimension B, sitting; dimension C, crawling and kneeling; dimension D, standing; and dimension E), walking, running, and jumping [11]. The scoring system is as follows: a score of zero is assigned if the individual is unable to start the activity, a score of 1 is assigned if the individual starts the activity, a score of 2 is assigned if the individual starts the activity but only completes half of it, and a score of 3 is assigned if the activity is completed. The scores for each dimension should be calculated separately by summing the scores for that dimension and dividing by the total score for that dimension [11].

In the end, the scores for each dimension should be multiplied by 100 to find the percentage that the child achieved in each dimension. Finally, the percentages for all dimensions should be added and divided by 5 (the number of dimensions evaluated), yielding the percentage that the child scored in the GMFM (dimension E). This method is based on the techniques of Luciana Ventura De Pina, PhD and Ana Paula Cunha Loureiro, PhD [12]. Both scales, the GMFM and the GMFCS, have been validated for use with children with CP. The GMFM assesses gross motor function and the GMFCS stratifies the functional level of children with CP. The Gross Motor Ability Estimator (GMAE) program will be used to analyze the GMFM results.

#### About PedsQL

The PedsQL is a multidimensional tool to measure health-related QoL in children and adolescents. It is a practical scale on the dimensions of health outlined by the World Health Organization, with questions related to the frequency of problems faced in the last month. The 23 item-PedsQL has 8 items for physical functioning; 5 for social functioning; 5 for emotional functioning; and 5 for school functioning, with 15 on patients’ psychosocial health.

### tDCS Intervention

#### Overview

The IG will receive 10 sessions of tDCS with a frequency of 5 daily sessions per week, over a period of 2 weeks. The stimulation will be administered during rehabilitation sessions. The electrodes will be 35-cm^2^ sponges moistened with normal saline (0.9% NaCl solution). The stimulation will consist of anodal stimulation in the primary motor cortex (M1) and cathodal stimulation in the supraorbital region on the contralateral side, at an intensity of 1 mA and a duration of 20 minutes at a steady-state level, with a ramp-up and ramp-down of 4 seconds. The stimulation will be combined with specific exercises in a circuit format to target dimensions D and E of the GMFM [13]. CG will undergo 10 sessions of sham tDCS associated with rehabilitation activities 5 times per week, for a total of 2 weeks. Activities that target dimension E of the GMFM will be organized as a circuit including running, walking, and jumping. Any change to the trial will be informed along with the reason for the change.

#### Safety

The device used, tDCS Soterix Medical (Soterix Medical Inc), model number 1300-A and serial number 13ITC0619005, is approved by the Food and Drug Administration for use as an experimental device and some for clinical purposes (ANVISA-80969860041). Further, 3.5-cm^2^ electrodes will be used. Bikson et al [13], prove if the purpose is to modulate neurophysiological measures for resting motor cortex stimulation in healthy young people (1 mA intensity), tDCS for 4 seconds induces acute excitability alterations.

Krishnan et al [14] demonstrated adequate safety of tDCS in children and adolescents. Well-accepted threshold of tDCS current density is <142.9 A/m^2^ or 14.29 mA/cm^2^. Each patient will undergo a thorough health assessment and medications in use will be registered as well as all health conditions. Cardiac frequency, blood pressure, temperature, respiration, clinical assessment of mental and functional status, and physical examination will be measured [15]. Any apparent side effects will be registered, and all changes to the study will be described so that individuals have the option to interrupt the trial; the trial may also be suspended.

#### Adverse Effects

Adverse effects will be assessed using the tDCS adverse effects questionnaire [16]. The dropout rate will be reported, and dropout reasons will be stated [15]. tDCS is generally well-tolerated with no serious adverse events [14]. The effects reported are rare, mild, and transient, with “redness,” “slight tingling,” “itching,” and “burning sensation” as the most reported events. The intensity of current, density, and electrode size will be optimized to modulate the tingling and itch perception during transcranial stimulation. Furthermore, we will not pass the well-accepted threshold of tDCS current density (<142.9 A/m^2^ or 14.29 mA/cm^2^) [14].

### Allocation

Participants will be randomly assigned to tDCS groups with a 1:1 allocation as per a computer-generated randomization schedule stratified by the patient’s age using 5 permuted blocks of 6 participants.

### Implementation

All participants will be randomized, and the randomization will be performed by the researcher who will carry out an intervention; the others will not know which group the participant is in. This member will upload the randomization results to the institution’s REDCap (Research Electronic Data Capture) database [17]. The researcher who will perform a physiotherapeutic intervention and the researcher who will apply the assessments and questionnaires will not have information about group allocation.

### Blinding

This will be a double-blinded study; during this study, the participants will be seen by three different teams: (1) the researcher who performs the assessment and questionnaires, (2) the neuromodulation team who apply the tDCS, and (3) the physiotherapist who performs the physiotherapy exercises. There will be no members working simultaneously on both teams. The results of randomization will only be available to the neuromodulation member responsible for the intervention, and the scales’ results will only be available to the researcher who performed the assessment and questionnaires. The results will be held in a different project in REDCap and will be available only to the researcher who makes the assessment and questionnaires.

### Emergency Unblinding

Emergency unblinding will be available under the determination of this study staff, in case of safety concerns related to the intervention.

### Interventions: Participants

Initially, we will explain to the child how the intervention process will be carried out. The electrodes will be moistened in saline solution and will be placed on the participant’s head, which may cause slight discomfort as it will wet the child’s head, and, to minimize this discomfort, we will frequently dry their face. Associated with this, the child will perform physical therapy exercises.

CGs for tDCS will receive placebo stimulation for 30 seconds, for 10 sessions to give the child an initial feeling of stimulation. Associated with the placebo, the child will perform physical therapy exercises, focusing on the demands of the GMFM dimension E. The tDCS intervention will receive a current of 1 mA, for 20 minutes, for 10 sessions also associated with physical therapy exercises, focusing on the demands of the GMFM dimension E.

The intervention lasts from 25 to 35 minutes with each participant and will have a researcher exclusively dedicated to it during the intervention period for data collection. There will be 5 sessions with each participant per week over 2 consecutive weeks, with sessions taking place on weekdays and not weekends. All included participants will complete 10 sections of physiotherapy with or without tDCS stimulation. The tDCS device contains 2 electrodes, an anode, and a cathode, in the form of 2 nonmetallic sponges measuring 5×5 cm^2^, moistened with saline solution. The anode will be positioned on the M1 of the participant’s dominant hemisphere, according to the 10-20 System EEG, while the cathode will be placed on the supraorbital region of the region contralateral to the anode. In the case of the experimental group, a current will be applied to M1 for 20 minutes for neurorehabilitation.

After 10 sessions, the GMFM test will be repeated with each patient along with the PedsQL. Further, 1 researcher will carry out the intervention or the placebo with tDCS and the other researcher will evaluate the results blindly, without knowing which group is the intervention and which is the control. The use of the equipment and the evaluation will be carried out in different rooms to guarantee the researchers’ blindness. Only the researcher in charge of using the tDCS will be aware of the allocation of children between experimental and CGs.

It is worth noting that the patients in the CGs learned, at the end of this study, the intervention that had the best effect on their motor function, respecting this study’s ethical perspective that all children should receive the best treatment.

## Results

There is a compelling need to tailor medical approaches more precisely, catering to individual differences. Achieving successful outcomes requires studies that use informative measures, robust evidence, and are applicable across diverse demographics.

In this proposal, we emphasize the importance of incorporating targeted and universally recognized outcomes, specifically focusing on motor skills impacted by the disorder. These skills are universally acknowledged and play a fundamental role in an individual’s daily life. Additionally, we are examining how these interventions influence the overall QoL for affected individuals. Data collection is ongoing and is expected to be completed by January 2025.

Moreover, our study aims to optimize treatment efficacy by exposing participants to multiple sessions. This strategy is designed to fine-tune the intervention’s impact, building upon a baseline established using the most credible rehabilitation practices available for this condition.

RCTs are not commonly conducted in pediatric populations, leading to a lack of dedicated information specific to this demographic’s unique needs and responses. Addressing this gap is crucial to better understand how interventions impact children with this condition.

## Discussion

A randomized controlled protocol using an additional tDCS was designed to improve specific motor skills and positively impact QoL in a homogeneous population with CP. This study aims to examine the impact of the combination of conventional rehabilitation exercises on the improvement of gross motor function in children with CP classified as level I and II in the GMFCS. The primary outcome was assessed using the scores of the GMFM dimension E and children’s QoL as the secondary outcome. tDCS seems to improve balance, gross motor function, and gait in children with CP [2,4-7]. Mixed and heterogeneous outcomes of varied motor skills made it difficult to understand who and in what conditions individuals may benefit from tDCS complementary use.

To address this issue, the specific protocol proposed focuses on a homogeneous population of children with CP who have preserved walking skills and will evaluate them for motor features and QoL. By using the GMFCS and GMFM to classify and map the skills and their changes, we aim to improve the evidence, since there is a clear outcome that can be measured, and optimize treatment for a personalized medicine approach in the heterogeneity of CP. Our goal is to gather evidence for a stratified population of children with lower locomotion restrictions, as suggested by de Almeida Carvalho Duarte et al [2], who highlighted the importance of studying more homogeneous populations in the field of CP to better understand gains in the functional independence of these children. This will allow us to know the treatment effect size and for whom it should be a good choice.

The costs and presence of adverse effects will be features considered to reveal the individual cost-effect balance [16], and dropout rates will indicate any potential reason for dropping out [15].

Stimulation has been done on the M1 motor area in children with GMFCS I, II, and III [18]. Children with GMFCS III compared with children classified as I and II present an important difference in the use of aid devices. Children with GMFCS levels I and II are very functional, running and walking independently and having fewer limitations, while children with GMFCS III need an assistive device, such as walker assistance. In places with ramps, they may not be able to go up and down, due to the difficulty in controlling their movements independently. In De Moura et al’s [4] meta-analysis, the difference in results was attributed to a high level of heterogeneity, potentially compromising the generalization of results and the understanding of who and in what conditions children would benefit from the use of tDCS. This protocol evaluated TDCS stimulation on the motor standardized measure and QoL outcomes.

Besides the efforts on RCT protocols, there are always the limits related to the artificial scenario observed in RCTs; many things are controlled and compared that differ from natural scenarios. However, the limitation in heterogeneity considering the differences in functional classification in motor skills may be an interesting strategy to decrease heterogeneity and truly understand the role of TDCS in the improvement of motor skills. Reporting quality, including sample size, control characteristics, important outcomes, and the methodology of intervention training and delivery may help in building a body of literature with better evidence [19,20].

QoL and health-related QoL are increasingly being considered therapeutic goals [19]. In the past decades, QoL has been defined as well-being across various domains and has become an important treatment goal, especially in diseases like CP [19]. Many studies have observed that parents report lower QoL for their children with CP than the children themselves report [19]. The emotional and social domains were evaluated equally for children and parents [19]. Furthermore, parents of children with severe impairment often report better QoL in the psychosocial domain [19]. Consistent findings suggest that children with CP can adapt well to their activity limitations and may have a satisfactory QoL despite deficits [19]. Improving QoL represents a crucial treatment objective for children with CP, necessitating precise measurement and targeted interventions aimed at achieving this outcome [19]. In conclusion, CONSORT-based report protocols based on homogeneous sampling and using relevant outcomes seem necessary to improve the evidence for personalized medicine, especially for the pediatric population.

## Abbreviations

CG: control group
CP: cerebral palsy
GMFCS: Gross Motor Function Classification System
GMFM: Gross Motor Function Measure
IG: intervention group
PedsQL: Pediatric Quality of Life Inventory
QoL: quality of life
RCT: randomized controlled trial
REDCap: Research Electronic Data Capture
tDCS: transcranial direct current stimulation
